# Biomarkers in biological fluids for dementia with Lewy bodies

**DOI:** 10.1186/s13195-014-0072-3

**Published:** 2014-10-16

**Authors:** Sebastian Schade, Brit Mollenhauer

**Affiliations:** 1 Paracelsus-Elena-Klinik, Klinikstraße 16, Kassel, D-34128, Germany; 2 Department of Clinical Neurophysiology, University Medical Center, Georg-August University, Robert-Koch Straße 40, Göttingen, 37075, Germany; 3 Department of Neurosurgery, University Medical Center, Georg-August University, Robert-Koch Straße 40, Göttingen, 37075, Germany; 4 Department of Neuropathology, University Medical Center, Georg-August University, Robert-Koch Straße 40, Göttingen, 37075, Germany

## Abstract

Dementia with Lewy bodies (DLB) has become the second most common neurodegenerative dementia due to demographic ageing. Differential diagnosis is still troublesome especially in early stages of the disease, since there is a great clinical and neuropathological overlap primarily with Alzheimer’s disease and Parkinson’s disease. Therefore, more specific biomarkers, not only for scientific reasons but also for clinical therapeutic decision-making, are urgently needed. In this review, we summarize the knowledge on fluid biomarkers for DLB, derived predominantly from cerebrospinal fluid. We discuss the value of well-defined markers (β-amyloid, (phosphorylated) tau, α-synuclein) as well as some promising ‘upcoming’ substances, which still have to be further evaluated.

## 1 Introduction

The isolation and successful detection of soluble β-amyloid (Aβ) from biological fluids in 1992 [1] revolutionized our knowledge of the connection between molecular pathology and cerebrospinal fluid (CSF) biomarkers. The progressing elucidation of the underlying and overlapping molecular pathologies of several neurodegenerative diseases, including Alzheimer’s disease (AD) and Parkinson’s disease (PD), has resulted in new biomarkers, which are urgently needed for more accurate diagnosis and as possible endpoints for clinical trials with future neuropreventive strategies. Thanks to vigorous defining criteria, the stratification of dementia with Lewy-bodies (DLB) as a molecular and clinical ‘in-between’ disorder has been pursued, but it still has clinical and neuropathological overlap with AD and PD [2],[3], which makes its early diagnosis difficult. While hallucinations in AD predict the coincidence of DLB with a specificity of 100% [4], the presence of non-motor symptoms, decreased dopamine transporter imaging [5] and response to dopaminergic therapy does not help to separate DLB from PD since only about 36% of subjects can be clinically classified as ‘responders’ using the L-dopa challenge [6],[7].

The quantification of Aβ1-42 in CSF in combination with total and/or phosphorylated tau protein was recently (together with positron emission tomography and structural magnetic resonance imaging) included in proposed research criteria for the clinical diagnosis of AD [8]. Here, reduced Aβ1-42 and increased total/phosphorylated tau protein in CSF correlate with neuropathologic features of the disease - that is, Aβ plaques and neurofibrillary tangles and neuronal loss - as well as clinical symptoms and disease intensity [9]. This CSF signature is a good predictor for cognitive decline in mild cognitive impairment [10] with high predictive value for identifying converters into overt dementia [11].

In PD, the underlying pathology is characterized by presynaptic α-synuclein (aSyn) aggregates and synapse rarefaction. Cognitive decline in PD occurs due to various reasons leading to destruction of essential networks [12]. The main question of whether and how much AD and aSyn pathology each contribute to cognitive decline in PD remains disputable [13]. A majority of DLB patients show increased cortical ^11^C-PIB binding, similar to AD [14],[15]. This suggests that DLB is actually a dementia associated with both aSyn and Aβ pathology, thereby possibly explaining its aggressive nature. PD with dementia (PDD), in contrast, shows a reduced prevalence of amyloid plaques and lower levels of cortical ^11^C-PIB binding than DLB [14]-[16]. This finding suggests that the dementia of PD subjects is more likely due to a specific aSyn pathology rather than only an overlap of other pathologies, in agreement with post-mortem observations [17],[18]. Others suggest, however, that the neuropathological correlate of PDD is a combination of different pathologies rather than the severity of any single pathology [3]. In addition, it has been proposed that the presence of Aβ triggers cognitive decline and dementia in PDD and DLB, but does not directly determine its nature [19]. In this context, it should be highlighted that incidental Aβ can be detected on occasion in healthy controls as well as in older subjects with PD [15], and decreased levels of CSF Aβ1-42 have been observed in recently diagnosed PD patients [20] and in patients with and without cognitive decline [21],[22]; this suggests that amyloid pathology does not have a single causative role in dementia. Furthermore, it has been shown that PD cases without dementia, but progression of cortical amyloid, show a faster cognitive deterioration than patients who are without Aβ deposits at baseline [23]. This is supported by a recent study that showed that low levels of CSF Aβ1-42 predict early-onset cognitive decline [24].

Thus, one current major problem is the overlapping neuropathology and the as yet incompletely understood molecular constituents of the pathological changes. It is expected that in the next years many more neuropathological entities will be identified and characterized at the molecular level, which will also influence our thinking of clinical phenotyping and the selection of biomarker candidates in the future [25].

## 2 Biomarkers in dementia with Lewy bodies

In addition to imaging biomarkers (see article by Mak and colleagues within this special series [26]), biomarkers in DLB include functional marker candidates, like electroencephalography slowing [27] and the detection of rapid eye movement sleep behaviour disorder and other sleep disturbances with polysomnography [28].

Dopamine transporter imaging studies are helpful in the differential diagnosis of AD, but are expensive and not widely available. A biological fluid marker would be more widely available (when shipped to a central laboratory), cheap, and have low safety concerns. Optimal marker candidates reflect a process proximal to the specific pathology; therefore, most studies on neurodegenerative disorders rely on marker candidates in the CSF. The ‘CSF analytic area’ comprises the area of the brain directly contributing to CSF composition, which incorporates the basal ganglia and the brainstem as main sites of interest in movement disorders. aSyn pathology in DLB (and PD) has also been shown in the periphery [29], however, which could enable the detection of a marker in peripheral biological fluids; for example, in blood or saliva [30]. So far, studies have been discrepant and need further validation (see below).

### 2.1 Cerebrospinal fluid biomarkers in dementia with Lewy bodies

The composition and alteration of CSF proteins, which might be disease-specific, underlines the value of CSF analysis as a diagnostic tool. Nevertheless, known and potential confounding factors need to be taken into account in any biological fluid study, such as protease activity, blood contamination - which occurs in 10 to 20% of lumbar punctures - and adhesion, especially of lipophilic proteins to certain external surfaces like polypropylene and glass. The adherence of standard operating procedures is essential to avoid false positive or negative findings.

### 2.2 Alzheimer’s disease biomarkers in dementia with Lewy bodies

The combination of decreased Aβ peptides and increased total/phosphorylated tau protein in CSF of AD subjects has shown diagnostic sensitivity and specificity above 80% in most studies [9].

Enzymatic cleavage of the 120 kDa transmembrane amyloid precursor protein leads to different fragments of the Aβ peptide [31]. Aβ seems to be important for the processing of information between neurons and is variably prone to aggregate and form plaques [32]. Amyloid plaques are found in the brain of patients with AD and DLB [31],[33] and contain primarily carboxy-terminally elongated forms of Aβ peptides, such as the fragment Aβ1-42.

As in AD, CSF levels of Aβ1-42 in DLB are regularly decreased compared to non-demented controls [34]. The correlation of decreased CSF Aβ values was shown by *in vivo* brain amyloid load in AD [35] but also appeared to be non-specifically decreased in other disorders without plaque pathology [36], which might be due to interindividual differences in the amount of amyloidogenic amyloid precursor protein processing. Attempts to normalize Aβ1-42 concentrations to Aβ1-40 (Aβ1-42/Aβ1-40 ratio) have been promising in terms of differentiating AD from DLB when compared with measuring these biomarkers individually [37]. Nevertheless, most studies could not define valuable cutoff scores to distinguish AD and DLB [38],[39], including one large autopsy study [40]. One reason might be the heterogeneity and possible interaction of neuropathological alterations in DLB. At least one study showed significantly lower CSF Aβ1-42 in DLB patients with senile plaques compared with DLB patients without senile plaques [41]. Another reason could be that a correlation between phosphorylated tau protein in CSF and its neuropathological equivalent (neurofibrillary tangles) was not found in patients with DLB [41].

Other fragments, isoforms and posttranslational modifications of Aβ peptides have also been proposed as CSF biomarkers for DLB. The oxidized version of Aβ1-40 (Aβ1-40^ox^), containing α-helical structures [42], has been shown to be increased in DLB patients compared with PDD patients and non-demented disease controls, which recently also has been shown in autopsy-proven AD and DLB [43]. This finding has been proposed to be a pathophysiological metabolism of Aβ1-40 specific to DLB, but needs to be replicated by independent groups and using alternative approaches. Other Aβ isoforms, such as Aβ1-37 and Aβ 1–38, are still a focus of research, but need to be better characterized [42]. Further posttranslational modifications (for example, fragmented forms of Aβ) possibly reflecting more disease-specific changes are currently been investigated by different groups [44].

The natively unfolded microtubule-associated phosphoprotein 68 kDa tau is important for the stabilization of microtubules [45]. Neuronal cells in AD contain pairwise helical protein filaments (neurofibrillary tangles) [46],[47] that are insoluble, stable polymers of the low molecular weight tau protein [48].

Intracellular tau protein is elevated in CSF of AD subjects and excessively increases in conditions with rapid neuronal loss - for example, Creutzfeldt-Jakob disease. In DLB, levels of CSF tau protein are lower compared to AD [40] and higher compared to PD and PDD [49]. Interestingly, patients with a diagnosis of ‘probable DLB’ according to the classification criteria [5] (which should be more accurate), tend to have even lower CSF tau protein levels [49].

Hyperphosphorylation of tau protein promotes its aggregation into neurofibrillary tangles. Some CSF studies have revealed better specificity for the discrimination of AD when using p-tau protein 181 rather than total tau protein [50]. Since the phosphorylation of tau protein in brain occurs to a lesser extent in DLB [51],[52], the quantification of phosphorylated tau species in CSF may serve as a specific marker to discriminate AD from DLB [50],[53]. Other phosphorylation sites of tau protein in CSF have been analyzed for their diagnostic value, showing similar results [54]-[57] (Table 1).

**Table 1:** Sumary of neuropathologic, clinical, imaging and fluid markers in dementia with Lewy bodies, Parkinson’s disease and Alzheimer’s disease

	**AD**	**AD and DLB**	**DLB**	**DLB and PD(D)**	**PD**
**Neuropathology**		Neurofibrillary tangles		Lewy bodies	
		Amyloid plaques			
**Clinical symptoms**		Cognitive decline	Spontaneous hallucinations	Cognitive decline	
				RBD	
				L-Dopa response	
**Nuclear imaging**		PIB binding↑		DAT↓	
**CSF biomarkers**	tau ↑	β-Amyloid 1–42 ↓	β-Amyloid 1-40^ox^↑	α-Synuclein↓	α-Synuclein↓
	p-tau ↑	Soluble NG2↓	CART↓	Neurosin↓	Oligomeric α-synuclein↑
			NF↑		
**Serum biomarkers**			H-FABP ↑		
			Ca/Mg↑		

AD, Alzheimer’ disease; CART, cocaine and amphetamine regulated transcript; CSF, cerebrospinal fluid; DAT, dopamine transporter imaging; DLB, dementia with Lewy bodies; H-FABP, heart-type fatty acid binding protein; NF, neurofilament; NG2, Neuron glia 2; PD, Parkinson’s disease; PDD, Parkinson’s disease with dementia; PIB, Pittsburgh compound B; p-tau, phosphorylated tau protein; RBD, REM sleep behaviour disorder.

### 2.3 Parkinson’s disease biomarkers in dementia with Lewy bodies

The 140 amino acid aSyn is predominantly expressed in the pre-synapses supporting the formation and transport of vesicles [58] and is the major constituent of Lewy bodies, the generally accepted pathological hallmark of PD and DLB, and it is also present in the glial cytoplasmic inclusions of multiple system atrophy [59],[60].

Full-length aSyn has been detected in extracellular biological fluids, including plasma, conditioned cell media and most recently saliva [61],[62]. The quantification of extracellular aSyn has been proposed as a potential biomarker for synuclein-related disorders: most investigators have shown a reduction of CSF total aSyn in the synuclein-related disorders PD, DLB and multiple system atrophy [63]-[65]. A rather small study, but one that strictly controlled several possible confounders (for example, blood contamination, diurnal variation, food intake, rostro-caudal CSF gradient, gender, age), showed contradictory results, with an increase of aSyn in DLB compared with healthy controls and AD patients [66]. These results need to be replicated, but possible confounding factors (for example, blood contamination, subject selection and technical/methodological differences, especially choosing the right antibodies to ensure accurate measurement of total aSyn rather than its fractions) should be even more rigorously taken into account when conducting further studies.

The underlying mechanism of decreasing CSF aSyn remains unclear to date, and could result from various scenarios, such as the reduction of aSyn release into the extracellular space due to intracellular aggregation; alteration of *SNCA* gene transcription [67], mRNA splicing [68] or protein processing [69]; a higher CSF flow with lower permeation of plasma aSyn into CSF; an enhanced clearance rate of aSyn from CSF [70]; or as yet unidentified factors or any combination of mechanisms [65]. Furthermore, aSyn might intracellularly aggregate in Lewy bodies and presynaptic terminals (thereby possibly decreasing the extracellular amount), since results from studies on aSyn in patients with AD have been somewhat heterogeneous, perhaps indicating a subgroup of AD patients with additional Lewy body pathology and a clear mismatch of high p-tau protein 181 and low aSyn CSF levels [71]. A possible explanation for increased CSF aSyn levels (in addition to the inverse of the mechanisms described above) might be that they partly reflect neuronal and/or axonal injury, which would be in line with a correlation of total tau values and aSyn in CSF samples of AD patients [71], although a correlation between aSyn levels and regional brain atrophy could not be detected [72].

Whereas methods for quantifying total aSyn detect mono- and oligomeric forms, an oligomer-specific aSyn assay has been established that uses the same monoclonal antibody for both capture and detection [73]. Oligomeric aSyn comprises up to 10% of the total aSyn content of CSF. Independent studies show an increase of CSF oligomeric aSyn in PD compared with AD, progressive supranuclear palsy and controls [73],[74]. Together with the reduced CSF total aSyn, the ratio of oligomeric to total aSyn had a sensitivity of 89.3% and a specificity of 90.6% for the diagnosis of PD in this study [74].

Further investigation of the specificity of the antibodies and total and oligomeric aSyn enzyme-linked immunoassay techniques are needed, as are independent studies on other posttranslationally modified aSyn species, studies quantifying CSF aSyn in longitudinal patient cohorts, as well as studies of aSyn in other biological fluids.

Neurosin, a protein suggested to cleave aSyn and thereby potentially with a major role in the pathomechanisms of diseases associated with aSyn pathology, was shown to be reduced in CSF of patients with synuclein-related disorders compared with healthy controls and patients with AD. The lowest levels have been found in patients with DLB, thereby offering a new option for a potential biomarker [75].

Other PD biomarkers in CSF have not yet been investigated in DLB, such as the multifunctional protein DJ-1 and its oxidized forms involved in many cellular processes [76]-[78], and other synaptic proteins.

### 2.4 Other potential biomarkers for dementia with Lewy bodies

#### 2.4.1 Neurofilaments

Neurofilaments (NFs) are involved in structural integrity and cell/organelle motility along the axons and determine axon calibre. CSF levels of NFs have been found to be elevated in DLB, but no significant differences have been observed in comparison with other dementias. Therefore, NFs seem to provide only a general hint of neuronal and axonal dysfunction without differential value for separating DLB from other disorders [79]. But data are still rare. In particular, subsets of NFs need to be evaluated further, since various types of neurons are affected in the different forms of dementia, perhaps meaning that different patterns of elevated NFs are potential biomarkers for differential diagnosis of dementias. Three different subunits of NF (light (NF-L), medium (NF-M) and heavy (NF-H)) have been defined. The filament is made up of one NF-L and either NF-M or NF-H arranged head to tail [80],[81].

#### 2.4.2 Fatty acid-binding proteins

Fatty acid-binding proteins (FABPs) are a family of small intracellular proteins that facilitate the transport of fatty acids between the cell membrane and different organelles [82]. Lower levels of heart-type FABP have been reported in brains from patients with Down’s syndrome and AD [83]. Serum FABP levels are elevated in a quite distinct manner in DLB [84],[85].

#### 2.4.3 Other potential biomarkers

On the basis of stronger pathological involvement of dopaminergic and serotonergic pathways in DLB than in AD, several neurotransmitters and their metabolites have been investigated. Reduced levels of the metabolites homovanillic acid, 5- hydroxyindolacetic acid and 3-methoxy-4-hydroxyphenylethyleneglycol have been found in DLB compared with AD [86]. Especially the latter, in combination with total tau protein, p-tau and Aβ1-42, could increase the sensitivity and specificity of discriminating those entities [87].

The chondroitinase sulphate proteoglycan Neuron glia 2 is a proteoglycan involved in several basic cellular mechanisms of pericytes as well as oligodentrocyte progenitor cells and its soluble form can be detected in CSF. Lower levels of soluble Neuron glia 2 have been found in CSF of patients with AD and DLB, but not in patients with PD or PDD, thereby implicating some kind of association with the accumulation of Aβ rather than aSyn. Results are preliminary and mechanisms far from being understood, but further investigations seem to be worthwhile [88].

Cocaine and amphetamine regulated transcript is a neuropeptide which is expressed selectively in the hypothalamus and was recently found to be present at significantly reduced levels in the CSF of DLB patients compared with controls and patients with AD [89]. Further studies are needed to confirm these preliminary data resulting from a rather small patient sample. Similarly, elevated levels of calcium and magnesium in CSF as well as of magnesium in blood were found by a Swedish study group, which used mass spectrometry to compare DLB patients with healthy controls and patients with AD [90]. These findings have to be replicated by independent groups. It is noteworthy that following our growing knowledge of molecular genetics in the field of neurodegenerative diseases, there have been high expectations that some gene products (for example, DJ-1, glucocerebrosidase) might be of use as biomarkers. Unfortunately, results have been either heterogeneous or rare in terms of DLB [91].

Finally, new diagnostic proteins might be discovered by proteomic studies. So far, some ‘protein peaks’ have been found as potential differential biomarkers, but these have either not been attributed to specific proteins [92] or have not been confirmed by further studies [93]. It is problematic that there is a lack of consistency across proteomic studies, which might be due to strong variations during sample preparation prior to the proteomic experiment itself (for example, degradation of proteins by storage material, contamination with blood) [94]. Therefore, standardized procedures are needed.

## 3 Conclusion

This review summarizes current studies on neurochemical marker candidates for DLB. Overall, it is clear that DLB is a disease ‘in between’ AD and PD, which is supported by clinical, imaging, neuropathological and neurochemical studies. Biomarker candidates from the AD and PD fields have been tested in DLB, but only a few have been shown to more specifically reflect the underlying DLB. Most of the markers reflect neuropathological features, but as long our discrimination of PDD and DLB is based only on an arbitrary ‘one-year rule’ without separation based on molecular pathology, biomarker studies with DLB subjects will be hampered [95].

## 4 Note

This article is part of a series on Lewy Body Dementia, edited by Ian McKeith and James Galvin. Other articles in this series can be found at http://alzres.com/series/LewyBodyDementia.

## Abbreviations

Aβ: β-amyloid

AD: Alzheimer’ disease

aSyn: α-synuclein

CSF: Cerebrospinal fluid

DLB: Dementia with Lewy bodies

FABP: Fatty acid-binding protein

NF: Neurofilament

PD: Parkinson’s disease

PDD: Parkinson’s disease with dementia

## Competing interests

The authors declare that they have no competing interests.
